# Regulating the proinflammatory response to composite biomaterials by targeting immunometabolism

**DOI:** 10.1016/j.bioactmat.2024.05.046

**Published:** 2024-06-07

**Authors:** Chima V. Maduka, Ashley V. Makela, Anthony Tundo, Evran Ural, Katlin B. Stivers, Maxwell M. Kuhnert, Mohammed Alhaj, Ehsanul Hoque Apu, Nureddin Ashammakhi, Kurt D. Hankenson, Ramani Narayan, Jennifer H. Elisseeff, Christopher H. Contag

**Affiliations:** a Comparative Medicine & Integrative Biology, Michigan State University, East Lansing, MI, 48824, USA; b Department of Biomedical Engineering, College of Engineering, Michigan State University, East Lansing, MI, 48824, USA; c Institute for Quantitative Health Science & Engineering, Michigan State University, East Lansing, MI, 48824, USA; d Translational Tissue Engineering Center, Johns Hopkins University School of Medicine, Baltimore, MD, 21231, USA; e Department of Chemical Engineering & Materials Science, Michigan State University, East Lansing, MI, 48824, USA; f Department of Biomedical Sciences, College of Dental Medicine, Lincoln Memorial University, Knoxville, TN, 37917, USA; g Department of Orthopedic Surgery, University of Michigan Medical School, Ann Arbor, MI, 48109, USA; h Department of Biomedical Engineering, Johns Hopkins University School of Medicine, Baltimore, MD, 21231, USA; i Department of Microbiology, Genetics & Immunology, Michigan State University, East Lansing, MI, 48864, USA

**Keywords:** Hydroxyapatite, Polylactide, Immunometabolism, Inflammation, Regenerative medicine

## Abstract

Composite biomaterials comprising polylactide (PLA) and hydroxyapatite (HA) are applied in bone, cartilage and dental regenerative medicine, where HA confers osteoconductive properties. However, after surgical implantation, adverse immune responses to these composites can occur, which have been attributed to size and morphology of HA particles. Approaches to effectively modulate these adverse immune responses have not been described. PLA degradation products have been shown to alter immune cell metabolism (immunometabolism), which drives the inflammatory response. Accordingly, to modulate the inflammatory response to composite biomaterials, inhibitors were incorporated into composites comprised of amorphous PLA (aPLA) and HA (aPLA + HA) to regulate glycolytic flux. Inhibition at specific steps in glycolysis reduced proinflammatory (CD86^+^CD206^-^) and increased pro-regenerative (CD206^+^) immune cell populations around implanted aPLA + HA. Notably, neutrophil and dendritic cell (DC) numbers along with proinflammatory monocyte and macrophage populations were decreased, and Arginase 1 expression among DCs was increased. Targeting immunometabolism to control the proinflammatory response to biomaterial composites, thereby creating a pro-regenerative microenvironment, is a significant advance in tissue engineering where immunomodulation enhances osseointegration and angiogenesis, which could lead to improved bone regeneration.

## Highlights

• Engineered immunomodulatory composite biomaterials reduce glycolytic activity in their microenvironment.
• Reduced glycolytic activity suppresses the proportion of proinflammatory immune cells.
• Leveraging immunometabolic strategies creates a pro-regenerative implant microenvironment.

## Graphical abstract

**Figure:**
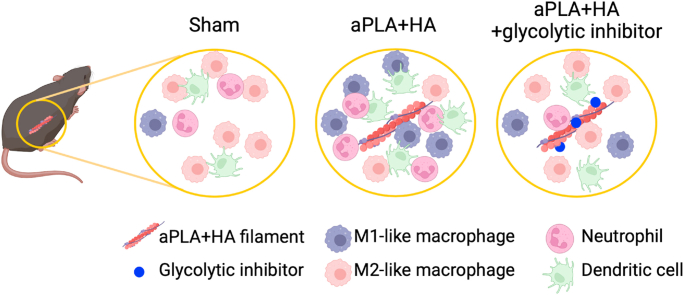

## 1 Introduction

Composite biomaterials comprising polylactide (PLA) and hydroxyapatite (HA) are often fabricated for clinical applications involving bone, cartilage and dental regenerative engineering. The combination of PLA with HA overcomes the brittleness of HA, while increasing the tensile modulus and hardness of the polymer to approximate that of trabecular bone [[1], [2], [3], [4], [5]]. Importantly, because 70 % (by weight) [6,7] of bone tissue is HA, HA is bioactive and can facilitate new bone [[8], [9], [10]], cartilage [11] and dental [12] tissue formation. Thus, HA confers osteoconductive characteristics to composite biomaterial formulations. Accordingly, it has been shown that inclusion of, or coating with, HA enhances bone-implant integration (osseointegration) [[13], [14], [15]]. In turn, enhanced osseointegration has been shown to reduce the risk of implant failure, and thus increase the longevity of total knee and hip replacements [[16], [17], [18], [19]].

Hydrolytic byproducts of PLA degradation were previously thought to elicit the foreign body response by reduced pH in the biomaterial microenvironment [20]. This notion originated from the correlation of decreased bioluminescence of the bacterium *Photobacterium phosphoreum* with reduced pH in unbuffered solutions containing PLA breakdown products [21]. However, recent advances now demonstrate that hydrolytic byproducts of PLA degradation activate surrounding immune cells by altering cellular bioenergetics and significantly increasing glycolytic flux (activity), resulting in metabolic reprogramming of the biomaterial microenvironment [22,23]. In a manner dependent on CCR2 and CX3CR1 signaling, immunometabolic cues regulate the trafficking of circulating monocytes to the PLA biomaterial microenvironment [24]. Consequently, targeting metabolic reprogramming using inhibitors such as aminooxyacetic acid (a.a.) or 2-deoxyglucose (2DG) effectively modulates the foreign body response to implanted PLA as demonstrated by reduced neutrophil recruitment, increased IL-4 production from T helper 2 cells and γδ+ T-cells, and skewing of monocyte, macrophage and dendritic cell populations toward pro-regenerative phenotypes [22,24]. These two inhibitors act at different steps in glycolysis; a.a. inhibits uptake of glycolytic substrates and glutamine metabolism, 2DG inhibits hexokinase in the glycolytic pathway [25,26].

Compared to PLA alone, the incorporation of HA exerts immunomodulatory effects by: a) decreasing the relative levels of proinflammatory (CD86^+^CD206^-^) dendritic cells (DCs), including proinflammatory DCs expressing class II major histocompatibility complex (MHC II); b) increasing the relative levels of transition (CD86^+^CD206^+^) and anti-inflammatory or pro-regenerative (CD206^+^) DCs, including those expressing MHC II; c) reducing the relative levels of proinflammatory monocytes and macrophages relative to transition cell populations in the biomaterial microenvironment [24]. Collectively, these immunomodulatory effects are able to enhance osseointegration and angiogenesis during skeletal tissue regeneration [9,14,27], with transition immune cells playing a crucial role [28,29]. However, HA alone [13,[30], [31], [32]] or as a composite with PLA [24,33] chronically activates neutrophils counteracting its beneficial immunological effects. Accordingly, short-term studies [34,35] are more likely to report beneficial immunomodulatory effects than long-term (>2 years) studies [36] when applying bulk composite implants comprising HA and PLA. Moreover, wear particles of HA (from coated metal implants applied in total knee and hip replacements) are known to drive chronic inflammation leading to implant failure [13,15], a process that is affected by particle size [32,37,38] and morphology [39]. In fact, HA wear particles have been shown to be recognized by the opsonin receptor [40] as well as by Toll-like receptor 4 [[15], [41]], upregulating proinflammatory genes [42], and activating both nuclear factor-kappa B and interferon regulatory factor 3 [41] to trigger the production of proinflammatory cytokines in a manner dependent on the membrane proximal kinase, *Syk*, as well as members of the mitogen-activated protein kinase family of signaling molecules [43]. In the bone microenvironment, this could drive osteolysis [36] by increasing osteoclastogenesis through upregulating M-CSF and RANKL [44].

Despite aforementioned *in vitro* and *in vivo* evidence that adverse immune responses could occur with HA and composites of HA with PLA, corresponding immunomodulatory strategies are yet to be explored. Furthermore, while the majority of prior studies have focused on the inflammatory underpinnings of HA particles [15,32,37,38,41,43,45], developing immunomodulatory strategies for bulk HA composites has not been addressed. Here, modulating glycolytic flux by incorporating glycolytic inhibitors into a composite material comprised of amorphous polylactide (aPLA) and HA (aPLA + HA) is demonstrated to lead to a pro-regenerative implant microenvironment. Inhibiting different glycolytic steps reduces the proportion of proinflammatory signals and increases the relative levels of pro-regenerative CD45^+^ immune cell population in the microenvironment surrounding implanted PLA-HA composite biomaterials. Notably, Ly6G^+^ neutrophil populations are decreased following the incorporation of glycolytic inhibitors in aPLA+HA composites. While overall levels of recruited CD11b^+^ monocytes and F4/80^+^ tissue macrophages do not decrease with the incorporation of glycolytic inhibitors in aPLA+HA composites, the respective proinflammatory proportions of these populations are reduced. In addition, dendritic cell populations are reduced by the incorporation of glycolytic inhibitors in implanted PLA-HA composites. Notably, Arginase 1 levels were increased in dendritic cells and dendritic cells expressing MHC II by incorporation of a glycolytic inhibitor. Control of inflammatory responses to biomaterial composites using glycolytic inhibitors is a significant advancement that could lead to enhanced osseointegration and angiogenesis by generating a pro-regenerative microenvironment resulting in improved tissue regeneration [9,14,46].

## 2 Result and discussion

Having observed the pivotal role that altered metabolism plays in the foreign body response to polylactide [[22], [23], [24]], it was hypothesized that locally modifying immunometabolic cues in the composite biomaterial microenvironment will modulate adverse inflammatory responses. To test this hypothesis, aPLA + HA biomaterials, with or without incorporated aminooxyacetic acid (a.a.) or 2-deoxyglucose (2DG) at previously optimized doses [[22], [23], [24]], were subcutaneously implanted into C57BL/6J mice. For reference, sham controls where incisions were made without biomaterial implantation were included.

**Fig. 1:**
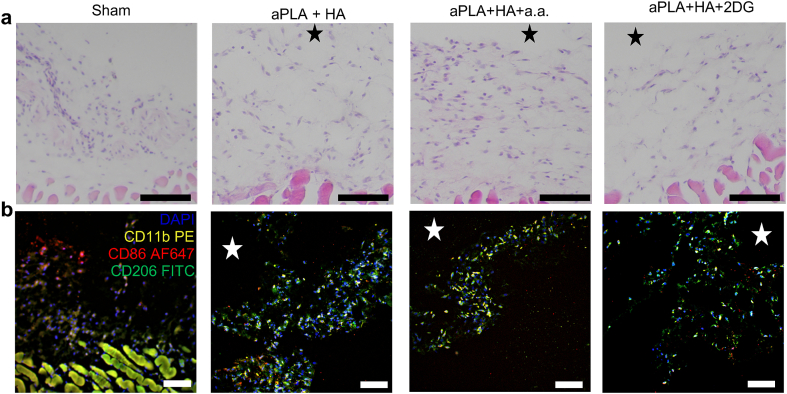
Histological evaluation of the amorphous polylactide-hydroxyapatite composite implant microenvironment with and without metabolic inhibitors suggests cellular infiltration. a, Hematoxylin and eosin staining reveals immune cellular infiltrates around implanted biomaterials (stars). b, Immunohistochemical staining using DAPI, CD11b-PE, CD206-FITC and CD86-AF647 suggests the presence of immune cells of varied immunophenotypic compositions. Scale bars, 100 μm, amorphous polylactide (aPLA), hydroxyapatite (HA), aminooxyacetic acid (a.a.), 2-deoxyglucose (2DG).

Preliminary observation by hematoxylin and eosin staining at 6 weeks after implantation revealed cellular infiltration in the vicinity of implanted biomaterials (Fig. 1a). Infiltrating cells expressed a variety of immune cellular markers, including CD11b, CD86 and CD206 (Fig. 1b), exciting curiosity and informing the need for more in-depth investigation. Accordingly, tissues around similarly-implanted biomaterials were harvested at 11 weeks for flow cytometry analyses. Having characterized the physiochemical properties and degradation profiles of various types of polylactides [22], aPLA was used for its faster degradation compared to semi-crystalline formulations to better model the inflammatory response in the duration chosen for the in-vivo study herein.

**Fig. 2:**
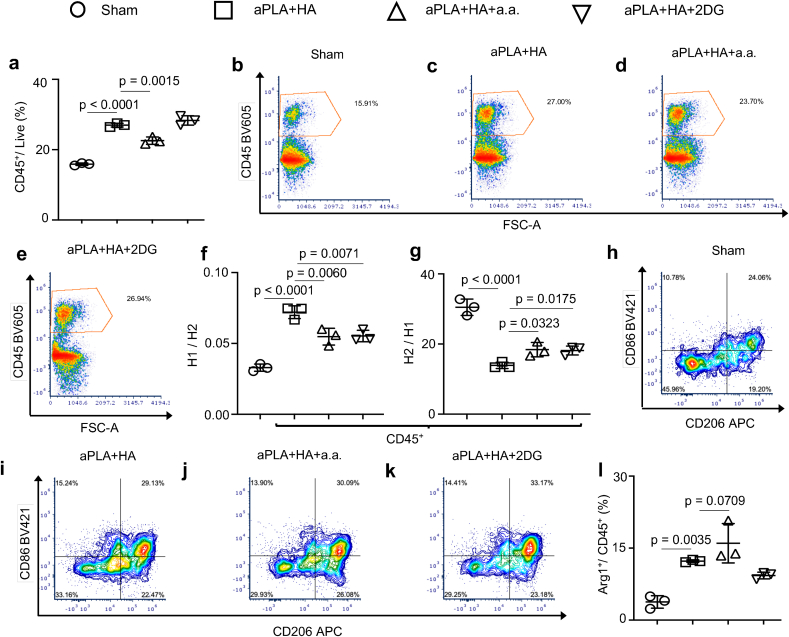
Glycolytic inhibition in the amorphous polylactide-hydroxyapatite composite biomaterial microenvironment modifies the numbers and inflammatory states of recruited nucleated hematopoietic cell populations. a-e, Flow cytometry quantification (a) and representative plots (b–e) of nucleated hematopoietic (CD45^+^) cells gated on live cells. f, Fold change of proinflammatory (H1; CD86^+^CD206^-^) cells with respect to anti-inflammatory (H2; CD206^+^) cells. g, Fold change of H2 with respect to H1 cells. h-k, Representative flow plots of CD86 and CD206 cells gated on CD45^+^ cells. l, Quantification of Arginase 1 (Arg1^+^) cells gated on CD45^+^ populations. One-way ANOVA followed by Tukey's or Newman-Keul's multiple comparison test, n = 3; amorphous polylactide (aPLA), hydroxyapatite (HA), aminooxyacetic acid (a.a.), 2-deoxyglucose (2DG).

Although implantation of aPLA + HA increased overall nucleated hematopoietic (CD45^+^) cell populations, incorporation of a.a. but not 2DG reduced CD45^+^ levels (Fig. 2a–e). To understand the relative levels of polarized CD45^+^ populations, proinflammatory subsets [47] were designated as CD86^+^CD206^-^ and anti-inflammatory subsets [46] as CD206^+^. Relative to sham controls, the fold change of proinflammatory CD45^+^ cells with respect to anti-inflammatory CD45^+^ cells was elevated (Fig. 2f). However, incorporation of either a.a. or 2DG reduced proinflammatory CD45^+^ proportions in comparison to aPLA+HA alone (Fig. 2f). Interestingly, implantation of aPLA + HA decreased the fold change of anti-inflammatory CD45^+^ cells with respect to proinflammatory CD45^+^ cells, likely due to the polylactide content [24] of the composite biomaterial. In contrast, incorporation of either a.a. or 2DG increased anti-inflammatory CD45^+^ levels compared to aPLA+HA only (Fig. 2g–k). Moreover, incorporating a.a. tended to increase the frequency of CD45^+^ cells expressing Arginase 1 (Arg 1^+^) even though this trend was not statistically significant (Fig. 2l).

**Fig. 3:**
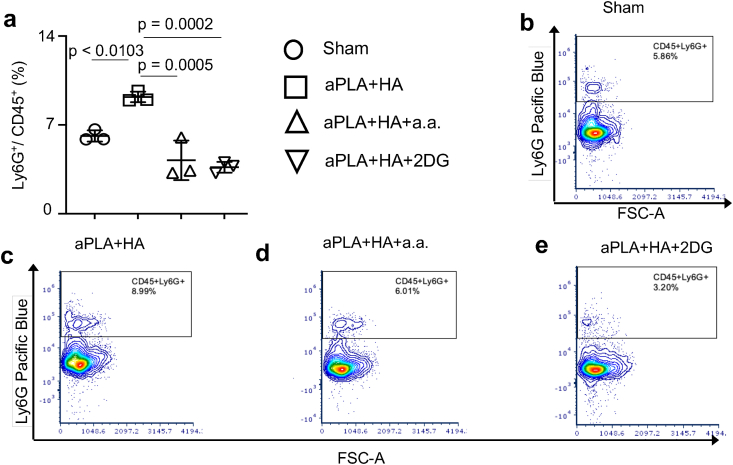
Incorporation of metabolic inhibitors modulate neutrophil recruitment in the amorphous polylactide-hydroxyapatite composite biomaterial microenvironment. a-e, Quantification (a) and representative flow cytometry plots (b–e) of neutrophils (Ly6G^+^) cells gated on CD45^+^ populations. One-way ANOVA followed by Tukey's multiple comparison test, n = 3; amorphous polylactide (aPLA), hydroxyapatite (HA), aminooxyacetic acid (a.a.), 2-deoxyglucose (2DG).

Previously, it was observed that, relative to aPLA alone, aPLA + HA does not reduce Ly6G^+^ neutrophils recruited to the biomaterial microenvironment [24]. Here, the findings illustrate that compared to sham controls, aPLA + HA implantation elevated neutrophil levels (Fig. 3a–e). Remarkably, incorporating a.a. or 2DG in aPLA + HA modulated this proinflammatory tendency (Fig. 3a–e). Elevated neutrophil levels are prevalent in murine bone defects implanted with micron-sized HA particles, an effect that is reduced by using nano-sized HA particles [32]. Reduced neutrophil levels are correlated with the pro-regenerative macrophage phenotype that is necessary to drive bone regeneration [32]. This observation is translationally relevant as HA potently activates human neutrophils *in vitro* [13,30,31].

**Fig. 4:**
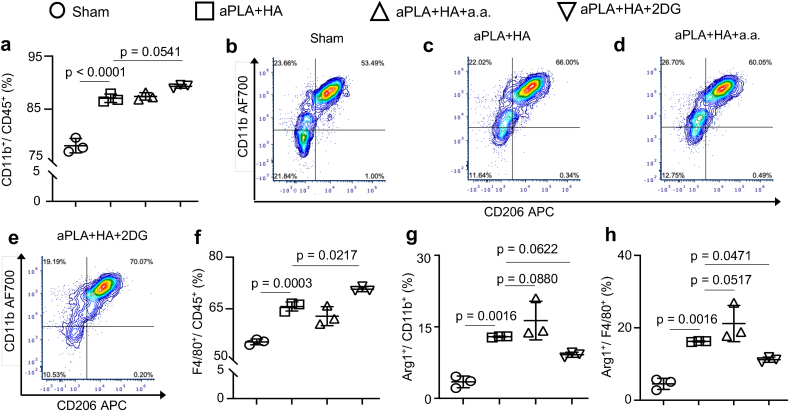
Monocyte and macrophage populations are differentially affected by targeting different glycolytic steps in the amorphous polylactide-hydroxyapatite composite biomaterial microenvironment. a, Flow cytometry quantification of monocytes (CD11b^+^ cells) gated on CD45^+^ populations. b-e, Representative plots of monocytes (CD11b^+^) and macrophages (F4/80^+^) gated on CD45^+^ populations. f, Quantification of macrophages in the composite biomaterial microenvironment. g-h, Quantification of Arginase 1 (Arg1^+^) monocytes (g) and macrophages (h). One-way ANOVA followed by Tukey's or Newman-Keul's multiple comparison test, n = 3; amorphous polylactide (aPLA), hydroxyapatite (HA), aminooxyacetic acid (a.a.), 2-deoxyglucose (2DG).

**Fig. 5:**
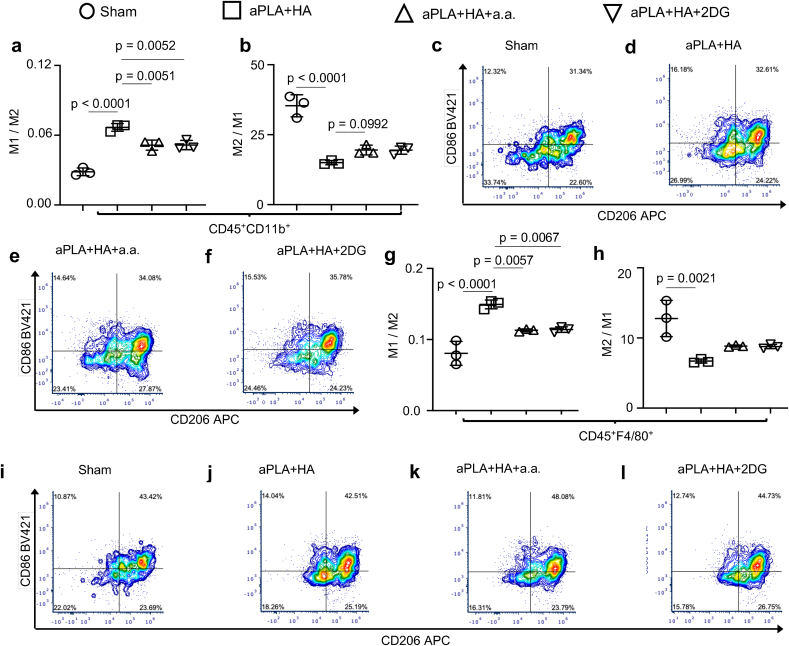
Activation states of monocytes and macrophages are modulated by glycolytic inhibition in the amorphous polylactide-hydroxyapatite composite biomaterial microenvironment. a, Fold change of proinflammatory (M1; CD86^+^CD206^-^) monocytes with respect to anti-inflammatory (M2; CD206^+^) monocytes. b, Fold change of M2 monocytes with respect to M1 monocytes. c-f, Representative plots of CD86 and CD206 cells gated on monocyte populations (CD45^+^CD11b^+^). g, Fold change of proinflammatory (M1; CD86^+^CD206^-^) macrophages with respect to anti-inflammatory (M2; CD206^+^) macrophages. h, Fold change of M2 macrophages with respect to M1 macrophages. i-l, Representative plots of CD86 and CD206 cells gated on macrophage populations (CD45^+^F4/80b^+^). One-way ANOVA followed by Tukey's multiple comparison test, n = 3; amorphous polylactide (aPLA), hydroxyapatite (HA), aminooxyacetic acid (a.a.), 2-deoxyglucose (2DG).

Next, the levels of CD11b^+^ monocytes [[48], [49], [50]] and F4/80^+^ macrophages [51] in the biomaterial microenvironment (CD11b is also expressed on some B-cells, neutrophils and macrophages [51]) were examined. Consistent with prior observations [33,52], implantation of aPLA + HA increased monocyte and macrophage proportions relative to sham controls, but incorporation of a.a. or 2DG did not reduce cellular recruitment (Fig. 4a–f). It was observed that aPLA + HA increased Arg1 levels among monocytes (Fig. 4g) and macrophages (Fig. 4h) relative to sham controls, likely from its immunomodulatory capability [9,32]. Additionally, incorporation of a.a. but not 2DG to aPLA + HA tended to further increase Arg1 levels among monocytes and macrophages, although this trend was not statistically significant (Fig. 4g and h). Relative to sham controls, aPLA + HA increased the fold change of proinflammatory monocytes with respect to anti-inflammatory monocytes; however, incorporation of either a.a. or 2DG reduced proinflammatory levels (Fig. 5a). Although implantation of aPLA + HA decreased the fold change of anti-inflammatory monocytes to proinflammatory monocytes, the tendency for a.a. to increase anti-inflammatory proportions was not statistically significant (Fig. 5b–f). Similar to observations made with monocytes, aPLA + HA elevated proinflammatory and reduced anti-inflammatory macrophage levels compared to sham controls (Fig. 5g–j). While incorporation of either a.a. or 2DG reduced proinflammatory macrophage levels, anti-inflammatory levels were not increased as observed in quantitated data and representative dot plots (Fig. 5g-l).

**Fig. 6:**
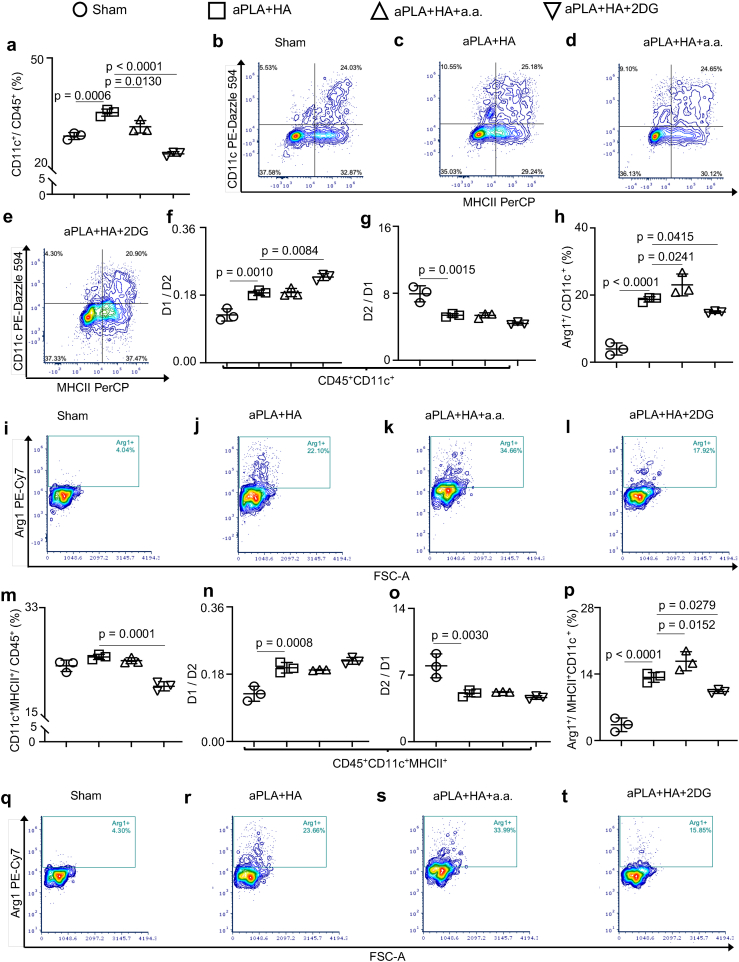
Proportions and inflammatory states of dendritic cells are affected in the amorphous polylactide-hydroxyapatite composite biomaterial microenvironment. a, Flow cytometry quantification of dendritic (CD11c^+^) cells gated on CD45^+^ cells. b-e, Representative plots of dendritic (CD11c^+^) cells with and without MHCII gated on CD45^+^ cells. f, Fold change of proinflammatory (D1; CD86^+^CD206^-^) dendritic cells with respect to anti-inflammatory (D2; CD206^+^) dendritic cells. g, Fold change of D2 with respect to D1 dendritic cells. h-l, Quantification (h) and representative plots (i–l) of Arginase 1 (Arg1^+^) dendritic cells. m, Dendritic cells expressing MHCII gated on CD45^+^ cells. n, Fold change of proinflammatory (D1; CD86^+^CD206^-^) dendritic cells expressing MHCII with respect to anti-inflammatory (D2; CD206^+^) dendritic cells expressing MHCII. o, Fold change of D2 with respect to D1 dendritic cells expressing MHCII. p-t, Quantification (p) and representative plots (q–t) of Arginase 1 (Arg1^+^) dendritic cells expressing MHCII. One-way ANOVA followed by Tukey's or Newman-Keul's multiple comparison test, n = 3; amorphous polylactide (aPLA), hydroxyapatite (HA), aminooxyacetic acid (a.a.), 2-deoxyglucose (2DG).

It was observed that CD11c^+^ dendritic cell populations were elevated in the aPLA+HA microenvironment compared to sham controls as previously reported [33,52], and that incorporation of either a.a. or 2DG reduced these dendritic cell numbers (Fig. 6a–e). Interestingly, compared to sham controls, the fold change of proinflammatory dendritic cells relative to anti-inflammatory dendritic cells was increased in the microenvironment of aPLA + HA implants; yet, incorporation of a.a. or 2DG did not reduce proinflammatory dendritic cell levels (Fig. 6f). Furthermore, although the fold change of anti-inflammatory dendritic cells to proinflammatory dendritic cells was decreased in aPLA + HA compared to sham controls, incorporating a.a. or 2DG did not increase anti-inflammatory dendritic cells proportions (Fig. 6g).

**Fig. 7:**
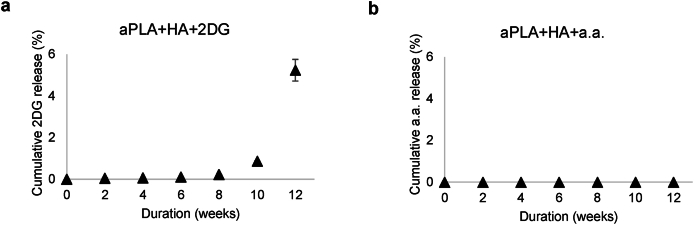
Release kinetics of glycolytic inhibitors suggests that only small amounts are released by 12 weeks. a-b, Whereas 5.23 % of 2-deoxyglucose (2DG; a) is released by 12 weeks, released aminooxyacetic acid (a.a.; b) levels are below detection limits of the applied mass spectrometry method. Mean (SD), n = 3, amorphous polylactide (aPLA), hydroxyapatite (HA).

Expression of Arg1 among dendritic cells was increased following implantation of aPLA + HA relative to sham controls (Fig. 6h–j). Notably, compared to aPLA + HA, incorporating a.a. further elevated Arg1 expression among dendritic cell populations (Fig. 6h-l). Dendritic cells expressing MHC II were similar between sham and aPLA + HA groups; incorporating 2DG decreased the numbers of dendritic cells expressing MHC II when compared to aPLA + HA (Fig. 6m). In comparison to sham controls, aPLA + HA both increased proinflammatory and decreased anti-inflammatory proportions of dendritic cells expressing MHC II (Fig. 6n-o), revealing previously unappreciated mechanisms by which composite biomaterials drive proinflammatory states. Compared to aPLA + HA only, incorporation of a.a. elevated Arg1 expression among dendritic cells expressing MHC II (Fig. 6p-t). Increased Arg1 expression in the composite biomaterial microenvironment could arise from inhibition of aspartate-aminotransferase by a.a., which obviates metabolic and transcriptional activation of immune cells into proinflammatory states [53]. Elevated Arg1 is a crucial driver of osteoinduction, creating a pro-regenerative composite biomaterial microenvironment [54]. Interestingly, observed immunomodulatory effects are likely the result of less than 6 % of released drugs by (Fig. 7a and b); the apparent absence of released a.a. is likely due to the inability of the utilized mass spectrometry technique to measure concentrations of a.a. that are <0.078 μM. Furthermore, drug release studies revealed that the total drug content (mean ± SD, n = 3) present in 200 mg of pellets was 131.51 ± 15.91 ng of a.a. or 243,070.90 ± 26,451.18 ng of 2DG.

In conclusion, the present study uncovers new ways by which composite biomaterials affect the immune microenvironment, such as altering the ratio of proinflammatory to anti-inflammatory CD45^+^ populations. Controlling metabolic states by modifying glycolytic flux around implanted composite biomaterials is capable of: a) decreasing neutrophil recruitment; b) decreasing proinflammatory monocyte and macrophage populations; c) decreasing dendritic cell numbers; d) and increasing Arg1 expression among dendritic cells and dendritic cells expressing MHC II. Aminoxyacetic acid (a.a.), one of the metabolic inhibitors, has already been used safely in clinical trials for the treatment of other disease conditions [55], making it a translatable inhibitor for incorporation into composite biomaterials for future clinical use. Prior to clinical translation, limitations of the current study that could be explored by future studies include the need to characterize the effects of implanting composite biomaterials containing embedded metabolic inhibitors in musculoskeletal tissues, such as bone defects, for regenerative medicine applications. Additional studies are needed to characterize longer *in vivo* time points as well as the effects of metabolic inhibitors on composite implants made from different biomaterials with varied physicochemical properties (e.g. crystallinity), which could impact immunomodulatory effects. Modulating the inflammatory responses to biomaterial composites by locally controlling the metabolism of immune cells around biomaterials represents an exciting advancement in the field that could significantly enhance osseointegration and angiogenesis by generating a pro-regenerative microenvironment [9,14,46].

## 3 Materials and methods

### 3.1 Metabolic inhibitors and their incorporation into biomaterials

The metabolic modulators 2-deoxyglucose (2DG; MilliporeSigma) and aminooxyacetic acid hemihydrochloride (a.a.; Sigma-Aldrich) were incorporated into composite biomaterials comprising amorphous polylactide (aPLA; PLA 4060D) and hydroxyapatite (HA; 2.5 μm particle sizes; Sigma-Aldrich) by melt-blending at 190 °C for 3 min in a DSM 15 cc mini-extruder (DSM Xplore). Based on prior *in vitro* studies [22], 189 mg of 2DG, 90 mg of a.a. and 200 mg of HA were compounded in 10 g of aPLA to approximate effective *in vivo* concentrations. Following extrusion from the DSM, a pelletizer (Leistritz Extrusion Technology) was used to create pellets. A second extrusion (Filabot EX2; 170 °C with air set at 93) into 1.75 mm diameter filaments was undertaken. Filaments were cut into 1 mm long sizes for implantation into mice and sterilized for 30 min by ultraviolet radiation.

### 3.2 Mouse studies

Female C57BL/6J mice were obtained from Jackson Laboratory. Whereas 8 week old mice were used for histological studies as previously reported [22], 9 week old mice were used for flow cytometry studies. Animal studies were approved by the Institutional Animal Care and Use Committee at Michigan State University (PROTO202100327). For the implantation of biomaterials, mice were anesthetized using 2–3% isofluorane mixed with oxygen. At the site of subcutaneous surgical implantation, the fur was shaved followed by disinfection of the skin using iodine and alcohol swabs. A pouch was made in the subcutis, followed by implantation of 1 mm long filaments of composite materials comprised of amorphous polylactide (aPLA) and HA (aPLA + HA), with and without incorporating a.a. and 2DG. Also included was a sham group with incision on the back of mice and pouch creation without biomaterial implantation. Each group comprised of n = 3 mice. In all cases, surgical glue (3 M Vetbond) was used to close the skin. Mice then received intraperitoneal or subcutaneous meloxicam (5 mg/kg) analgesia as well as postoperative saline.

### 3.3 Tissue histology and imaging

As previously described [22], six weeks following implantation, mice were shaved around the implanted biomaterial site (or sham site), then euthanized for excision of full skin tissues. Skin tissue biopsies were fixed in 4 % paraformaldehyde (PFA) overnight followed by cryopreservation by serial submersion in 10 %, 20 % and 30 % sucrose. Afterwards, tissues were frozen in optimal cutting temperature compound (Fisher HealthCare, USA) and sectioned using a cryostat (8 μm sections) and placed on a slide. Samples underwent routine hematoxylin and eosin staining followed by imaging using a Nikon Eclipse Ci microscope equipped with a Nikon DS-Fi3 camera (Nikon, Tokyo, Japan) for color acquisition and NIS elements BR 5.21.02 software.

For immunohistochemistry, slides were submerged in deionized water for 5 min followed by blocking with 1 % BSA in 1X PBS for 30 min. Sections were then incubated with PE anti-mouse/human CD11b (1:100, Biolegend cat#101208), AF647 anti-mouse CD86 (1:100, Biolegend cat#105020) and FITC anti-mouse CD206 (1:100, Biolegend cat#141703) overnight at 4 °C. Sections were then washed and treated with TrueVIEW Autofluorescence Quenching Kit with DAPI (Vector Laboratories, cat#SP-8500-15), as recommended by manufacturer. Imaging was performed on a Leica Dmi8 Thunder microscope equipped with a DFC9000 GTC sCMOS camera and LAS-X software (Leica, Wetzlar, Germany) using the lasers and filters: 395, open (DAPI), 475, 535/70 (FITC), 555, open (PE) and 635, open (AF647). Images were prepared using FIJI (ImageJ; V2.9.0).

### 3.4 Tissue digestion protocol for flow cytometry

Eleven weeks following implantation, mice were shaved around the implanted biomaterial site (or sham site), then euthanized for excision of tissue. Circular biopsies (8 mm diameter) were collected from each mouse and tissues were pooled from the same groups. Tissues were cut with surgical scissors for ∼1 min followed by digestion in an enzyme cocktail containing 0.5 mg/mL Liberase (Sigma-Aldrich), 0.5 mg/mL Collagenase Type IV (Stem Cell Technologies), 250 U/mL Deoxyribonuclease I (Worthington Biochemical Corporation) in 25 mM HEPES buffer (Sigma-Aldrich). The tissue/enzyme cocktail was incubated at 37 °C with 5 % CO2 on top of an orbital shaker, shaking at 70 rpm for 1 h. Following incubation, 5 mL of the tissue/enzyme cocktail mixture was run through a 70 μm filter into a 50 mL conical tube and the remaining tissues which were not digested were mechanically dissociated against the serrated portion of a petri dish. The resultant mixture was filtered into the previous 50 mL conical tube. Remaining undigested tissue in the 70 μm filter was again mechanically dissociated with the thumb press of a syringe plunger for optimal extraction of cells. The petri dish was washed with cold Hanks' Balanced Salt Solution without calcium, magnesium and phenol red (ThermoFisher Scientific), followed by filtration into the conical tube. Cells were centrifuged at 350G for 10 min followed by automated counting (Countess Automated Cell Counter, Invitrogen) for flow cytometry.

### 3.5 Flow cytometry

For flow cytometry staining in a polypropylene 96-well round bottom plate (Sigma, cat#P6866), 1 × 10^6^ cells were used. All staining steps were performed in 100 μL volume in the dark at 4 °C. Samples were first incubated with LIVE/DEAD Fixable Blue Dead Cell Stain kit (1:500, Thermofisher, cat#L23105) for 20 min. Thereafter, cells were washed once with flow buffer, followed by incubation with TruStain FcX (anti-mouse CD16/32) Antibody (BioLegend, Cat#101319; 1 μg/sample) in 50 μL volume for 10 min. The following antibodies were mixed together at 2x concentration in 50 μL and added directly to the cell suspension: BV605 CD45 (1:500, Biolegend, cat#103139), AF700 CD11b (1:300, Biolegend, cat#101222), BV785 F4/80 (1:300, Biolegend, cat#123141), BV421 CD86 (1:200, Biolegend, cat#105031), APC CD206 (1:200, Biolegend, cat#141707), PerCP MHCII (1:200, Biolegend, cat#107623), PacBlue Ly6G (1:250, BD Bioscience, cat#127611) and PE-Dazzle 594 CD11c (1:500, Biolegend, cat#117347). Cells and antibody mixture were incubated for 30 min. Cells were washed once prior to fixation and permeabilization (BD Cytofix/Cytoperm kit, cat#BDB554714) as per manufacturer's instructions. Cells were then resuspended in BD Perm/wash buffer with PE-Cy7 Arg1 (1:100, ThermoFisher, cat#25-3697-80). Cells were incubated with antibody mixture for 30 min. Cells were washed twice with BD Perm/wash buffer followed by resuspension in a final volume of 100 μL for flow cytometry analysis.

The Cytek Aurora spectral flow cytometer (Cytek Biosciences, CA, USA) was used to analyze samples. Furthermore, fluorescence minus one (FMO) samples guided gating strategies, and the software FCSExpress (DeNovo Software, CA, USA) was used to analyze flow cytometry data.

### 3.6 Drug release studies

To appraise release profiles of 2DG and a.a. from aPLA + HA composites, 200 mg of pellet (about 1 mm-long) was suspended in 1 mL of Milli-Q water (n = 3) at 37 °C in an orbital shaker set at 250 rpm for 12 weeks using a previously validated method [24]. Releasate (supernatant) was retrieved every 2 weeks, then replaced with 1 mL of Milli-Q water. Following the 12-week time point, undissolved pellet was suspended in 1 mL chloroform (Thermo Fisher Scientific) for complete dissolution. Thereafter, 0.5 mL Milli-Q water was added to partition off chloroform, while dissolving the water-soluble 2DG and a.a. After vigorously shaking the mixture, samples were centrifuged at 500 rpm for 3 min to separate the inorganic and organic phases, allowing the aqueous phase to be decanted and stored at −20 °C. The cumulative release plots (Fig. 7) were made to account for remnant 2DG and a.a. present in undissolved pellets. The total amount was calculated as the cumulative amount of drug released over the 12-week duration (n = 3).

Levels of 2DG and a.a. were measured by Liquid Chromatography-Electrospray Ionization Mass Spectrometry (LC-ESI-MS) as previously described [24]. Amounts of a.a. were measured using a Xevo TQ-S micro Triple Quadrupole Mass Spectrometer (Waters) interfaced with a Thermo Vanquish UPLC. 5 μL of sample was injected into the Waters Acquity HSS T3 column (2.1 × 100 mm, 1.8 μm) and a.a. was separated using the following gradient: phase A (10 mM Perfluoroheptanoic acid (PFHA) in water) and phase B (acetonitrile). On the other hand, 2DG was evaluated by a Xevo TQ-XS Triple Quadrupole Mass Spectrometer (Waters) interfaced with a Thermo Vanquish UHPLC. With 2DG, 5 μL of sample was injected into the Waters Acquity BEH-Amide column (2.1 × 100 mm, 1.7 μm) at a column temperature of 40 °C. The gradient used to separate 2DG was phase A (10 mM Ammonium Acetate in Water) and phase B (10 mM Ammonium Acetate in 90:10 Acetonitrile-Water). The column flow rate was 0.3 mL/min for 2DG and a.a., and ions were generated by electrospray ionization in positive mode (a.a.) or negative mode (2DG). Data were evaluated using the TargetLynx tool in the Waters MassLynx (v 4.2) software.

### 3.7 Statistics

GraphPad Prism was used as software (GraphPad Prism Version 9.5.1) for statistical data analysis. Results are presented as mean ± standard deviation (SD), with figure legends showing exact statistical test, p-values and sample sizes.

## Ethics approval and consent to participate

Animal studies were approved by the Institutional Animal Care and Use Committee at Michigan State University (PROTO202100327).

## Data availability

The data supporting the findings of this study are available within the paper and its Supporting Information.

## CRediT authorship contribution statement

**Chima V. Maduka:** Writing – review & editing, Writing – original draft, Visualization, Validation, Resources, Project administration, Methodology, Investigation, Data curation, Conceptualization. **Ashley V. Makela:** Writing – review & editing, Writing – original draft, Visualization, Validation, Software, Methodology, Investigation, Data curation. **Anthony Tundo:** Writing – review & editing, Methodology, Investigation. **Evran Ural:** Writing – review & editing, Methodology, Investigation. **Katlin B. Stivers:** Writing – review & editing, Methodology, Investigation. **Maxwell M. Kuhnert:** Writing – review & editing, Methodology, Investigation. **Mohammed Alhaj:** Methodology, Investigation. **Ehsanul Hoque Apu:** Writing – review & editing. **Nureddin Ashammakhi:** Writing – review & editing. **Kurt D. Hankenson:** Writing – review & editing, Supervision. **Ramani Narayan:** Writing – review & editing, Supervision. **Jennifer H. Elisseeff:** Writing – review & editing, Supervision. **Christopher H. Contag:** Writing – review & editing, Supervision, Resources, Project administration, Conceptualization.

## Declaration of competing interest

The authors declare no competing interest.
